# Dendrimer-Based N-Acetyl Cysteine Maternal Therapy Ameliorates Placental Inflammation *via* Maintenance of M1/M2 Macrophage Recruitment

**DOI:** 10.3389/fbioe.2022.819593

**Published:** 2022-01-28

**Authors:** Yang Liu, Quan Na, Jin Liu, Anguo Liu, Akosua Oppong, Ji Yeon Lee, Anna Chudnovets, Jun Lei, Rishi Sharma, Sujatha Kannan, Rangaramanujam M. Kannan, Irina Burd

**Affiliations:** ^1^ Integrated Research Center for Fetal Medicine, Department of Gynecology and Obstetrics, Johns Hopkins University School of Medicine, Baltimore, MD, United States; ^2^ Center for Nanomedicine, Department of Ophthalmology, Johns Hopkins University School of Medicine, Baltimore, MD, United States; ^3^ Department of Anesthesiology and Critical Care Medicine, Johns Hopkins University School of Medicine, Baltimore, MD, United States

**Keywords:** macrophage, M1/M2, placental inflammation, maternal therapy, DNAC

## Abstract

Intrauterine inflammation (IUI) is the primary cause of spontaneous preterm birth and predisposes neonates to long-term sequelae, including adverse neurological outcomes. N-acetyl-L-cysteine (NAC) is the amino acid L-cysteine derivative and a precursor to the antioxidant glutathione (GSH). NAC is commonly used clinically as an antioxidant with anti-inflammatory properties. Poor bioavailability and high protein binding of NAC necessitates the use of high doses resulting in side effects including nausea, vomiting, and gastric disruptions. Therefore, dendrimer-based therapy can specifically target the drug to the cells involved in inflammation, reducing side effects with efficacy at much lower doses than the free drug. Towards development of the new therapies for the treatment of maternal inflammation, we successfully administered dendrimer-based N-Acetyl Cysteine (DNAC) in an animal model of IUI to reduce preterm birth and perinatal inflammatory response. This study explored the associated immune mechanisms of DNAC treatment on placental macrophages following IUI, especially on M1/M2 type macrophage polarization. Our results demonstrated that intraperitoneal maternal DNAC administration significantly reduced the pro-inflammatory cytokine mRNA of *Il1β* and *Nos2*, and decreased CD45^+^ leukocyte infiltration in the placenta following IUI. Furthermore, we found that DNAC altered placental immune profile by stimulating macrophages to change to the M2 phenotype while decreasing the M1 phenotype, thus suppressing the inflammatory responses in the placenta. Our study provides evidence for DNAC therapy to alleviate IUI *via* the maintenance of macrophage M1/M2 imbalance in the placenta.

## Introduction

Intrauterine inflammation (IUI) is the primary cause of spontaneous preterm birth and predisposes neonates to long-term sequelae, including adverse neurological outcomes, and accounting for approximately 35% of neonatal deaths in 2017 (Chawanpaiboon et al., 2019; Hug et al., 2019). The pro-inflammatory cytokines induced by IUI lead to fetal inflammatory response syndrome (FIRS), which results in these neonatal complications (Romero et al., 2005; Burd et al., 2012; Cappelletti et al., 2016). There is currently a lack of treatment options for spontaneous preterm birth, and new avenues for treatment should not only address spontaneous preterm birth but also pediatric outcomes.

Pro-inflammatory stimulation causes changes in the maternal immune system and placental microenvironment, a process in which placental macrophages may play an important role. The macrophage is the primary cell in the acute inflammatory response. Generally, macrophages are categorized into two main phenotypes based on their function (Wang et al., 2019; Yunna et al., 2020); M1 macrophages are pro-inflammatory, while M2 macrophages are anti-inflammatory (An et al., 2017). In normal pregnancy, maternal decidual and fetal placental macrophages (also known as Hoffbauer cells) are maintained in an anti-inflammatory state to achieve immune tolerance of the fetus (Bolton and Bilbo, 2014).

*N*-acetyl-*L*-cysteine (NAC) is the amino acid L-cysteine derivative and a precursor to the antioxidant glutathione (GSH). NAC is commonly used clinically as an antioxidant with anti-inflammatory properties (Smilkstein et al., 1988). A clinical study found that oral NAC administration can reduce recurrent preterm labor in patients with a history of bacterial vaginosis (Shahin et al., 2009). In another double-blinded trial, in which NAC was used as a neuroprotective agent in the case of maternal chorioamnionitis, administration of NAC improved maternal inflammatory cytokines (Jenkins et al., 2016). However, side effects from the necessary high dosage, include nausea, vomiting, and gastric disruptions (Copersino et al., 2006; Shahin et al., 2009). Therefore, dendrimer-based therapy has been used to decrease the dose necessary to contain the inflammation.

Dendrimers ( ∼ 4 nm) are emerging as a promising strategy for targeted intracellular drug delivery. We have previously shown that dendrimer-drug conjugates specifically target activated macrophages and microglia in pre-clinical models of inflammation (Nance et al., 2016; Zhang et al., 2016; Nance et al., 2017; Nemeth et al., 2017). Dendrimer-drug conjugates offer many advantages over free drugs, including sustained/targeted delivery for improved efficacy and reduced drug side effects. We have also shown that polyamidoamine (PAMAM) dendrimers when conjugated to NAC (DNAC) specifically delivers NAC intracellularly into activated microglia/macrophages in the brain and is more effective than the free drug (Niño et al., 2018; Turk et al., 2018; Zhang et al., 2020). In an animal model of IUI, DNAC reduces preterm birth and perinatal inflammatory response (Lei et al., 2017). However, the mechanisms of action of DNAC in placenta have not been studied.

In this study, we sought to identify the immune mechanisms of DNAC in the placenta following IUI. We hypothesized that maternally administered DNAC reduces placental inflammation following IUI, through the maintenance of immune response, especially on macrophage recruitment and M1/M2 phenotypes and polarization.

## Materials and Methods

### Animal Preparation and Experimental Groups

All animal care and treatment procedures were approved by the Animal Care and Use Committee of Johns Hopkins University Hopkins‐IACUC Protocol No. MO14M326. CD-1 pregnant dams from Charles River were used for this study. Pregnant dams were subjected to a well‐established model of IUI as per a previously described protocol on embryonic day 17 (E17) (Burd et al., 2010; Elovitz et al., 2011; Dada et al., 2014; Lei et al., 2017; Lei et al., 2018; Liu et al., 2021; Na et al., 2021). Briefly, mice were anesthetized with isoflurane (Baxter # NDC 10019‐360‐60) before undergoing a mini‐laparotomy. After dressing the abdominal area, a 1.5‐cm midline incision was performed in the lower abdominal wall. Mice were randomized to intrauterine injections of either lipopolysaccharide (LPS), a model of IUI, or phosphate‐buffered saline (PBS) at E17. The dose of 25 μg of LPS (*E. coli* O55:B5, Sigma‐Aldrich, St Louis, MO, United States, *n* = 150) in 100 μl PBS or vehicle only (PBS, *n* = 38) was injected between the first and second embryos of the lower right uterine horn. Routine laparotomy closure was performed, and dams were returned to cages individually. One hour after surgery, dams were randomly allocated to additional treatments: DNAC or PBS. 10 mg/kg DNAC (Sigma‐Aldrich) in 100 µL PBS was injected intraperitoneally (IP), PBS was injected IP as vehicle. In total, four groups were utilized: PBS + PBS (*n* = 20), LPS + PBS (*n* = 96), LPS + DNAC (*n* = 54) and PBS + DNAC (*n* = 18). Pregnant mice were sacrificed 3, 6 or 12 h after the LPS injection to collect tissue samples of placenta for biochemical and histological analysis. Placentas were from the first four gestational sacs of the right uterine horn.

### Flow Cytometry

**FIGURE 1:**
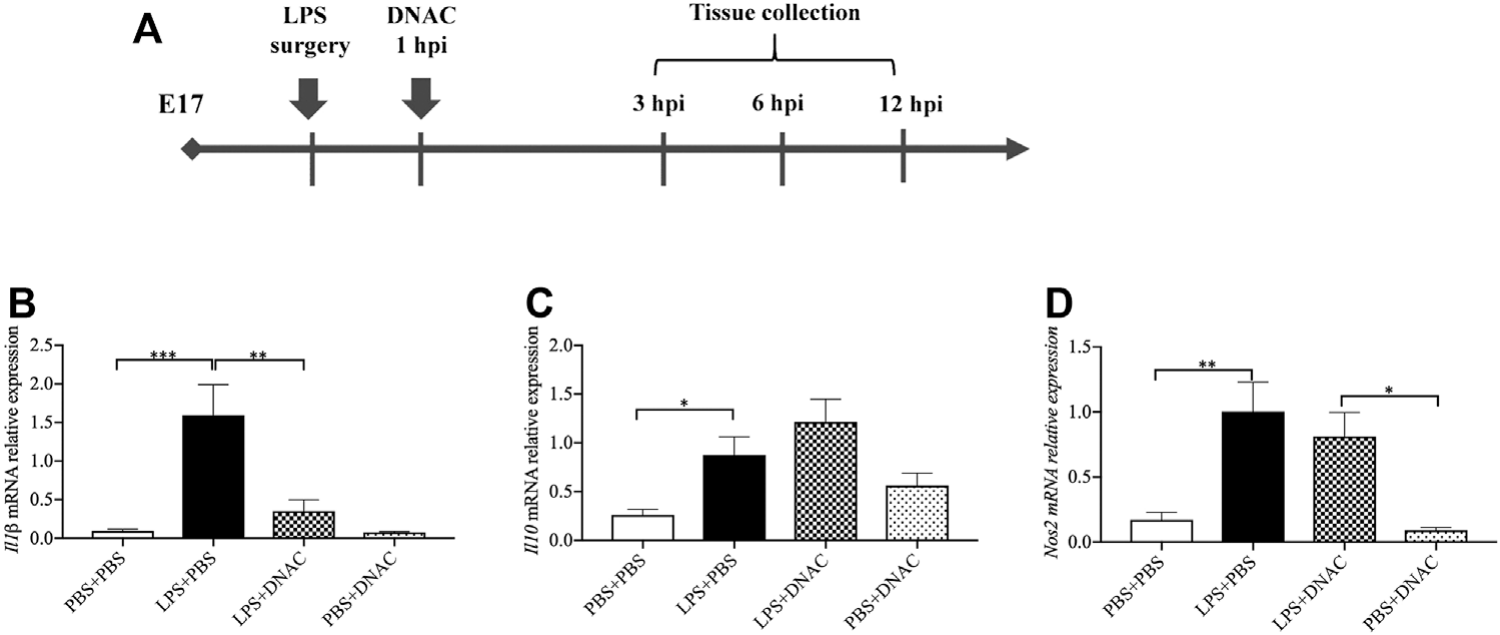
Measurements of inflammatory cytokine expression at mRNA level in placenta following exposure to intrauterine inflammation and maternal Dendrimer-based N-Acetyl Cysteine (DNAC) treatment. **(A)** Study Strategy. At embryonic (E) day 17, pregnant CD-1 mice underwent a mini-laparotomy in the lower abdomen for intrauterine injection of lipopolysaccharide (LPS). One hour later, dams received intraperitoneally injection of DNAC or phosphate-buffered saline (PBS). At 6 h post injection (hpi) dams were sacrificed. Placentas were harvested from each dam and quantitative polymerase chain reaction (QPCR) was performed to evaluate the effect of DNAC treatment. The mRNA levels of *Il1β* **(B)**, *Il10* **(C)** and *Nos2* **(D)** were statically compared between groups. PBS + PBS, *n* = 8; LPS + PBS, *n* = 8; LPS + DNAC, *n* = 7; PBS + DNAC, *n* = 6. *p < 0.05 **p < 0.01 ***p < 0.001. Data were reported as Mean ± SEM.

Placentas were harvested from the first gestational sacs in the right uterine horn, which is the most adjacent to the injection point, after 3, 6 or 12 h of surgery, respectively (Figure 1A). The decidual zone was further removed from the placenta. Single cells were prepared from placentas by manual and enzymatic (collagenase D, Roche, Indianapolis, IN, United States) digestion and then passed through a 70‐μm nylon mesh (BD Falcon cell strainer). Red blood cell lysis (ACK lysing buffer, ThermoFisher Scientific) and total cell counts were performed prior to surface staining. Isolated cells were stained with surface markers, at 1:100 concentration, in fluorescence activated cell sorting (FACS) buffer with 1 mM EDTA (Quality Biological, Gaithersburg, MD, United States) for 30 min at 4°C in the dark. OneComp eBeads (eBioscience) were used for compensation.

Antibodies and corresponding isotype are listed in Table 1. Data were acquired on an Attune™ NxT Acoustic Focusing Cytometer (ThermoFisher Scientific Invitrogen™, 2017) and analyzed with FlowJo version 10.1 (FlowJo, LLC, Ashland, OR, United States). Seven‐color flow cytometry was used to identify CD45^+^ leukocytes and macrophage populations. Debris and doublets were excluded by sequential gating on forward scatter height versus forward scatter area. They were then gate on CD45^+^ to identify all CD45^+^ leukocytes, gated on CD11b^+^ F4/80^+^ from CD45^+^ cells to identify placental macrophages, and gated on CD11b^+^ F4/80^+^ CD163^+^ to identify M2 placental macrophages.

**TABLE 1:** Antibody selection.

Antibody	Clone	Manufacture
Mouse anti-CD45	30-F11	BD
Mouse anti-CD11b	M1/70	BD
Mouse anti-F4/80	MF48028	ThermoFisher Scientific
Mouse anti-CD163	MR6F3	ThermoFisher Scientific

### RNA Extraction and Quantitative Polymerase Chain Reaction

After euthanizing the mice 6 h after the LPS or PBS injection, quantitative polymerase chain reactions (qPCR) were completed based on protocols described in the previous study. Placentas were isolated from the first through fourth gestational sacs of the right uterine horn and fresh frozen on dry ice, followed by long‐term storage at −80°C. Initially for each placental specimen, 2 µg of RNA was used for complementary 3) DNA synthesis in a 40 µL reaction, using Bio‐Rad iScript™ cDNA Synthesis Kit (Bio‐Rad, Cat. No. 170‐8891). The primers for actin (Assay ID Mm. PT. 39a.22214843. g), interleukin (IL)‐1β (Mm.Pt.58.44004828), IL-10 (Mm.Pt.58.13531087) and Nos2 (Mm.Pt.58.43705194) were obtained from Integrated DNA Technologies. The primer for 18S (Cat. No. 4310893E) was obtained from Applied Biosystems (Thermo Fisher Scientific).

### Histochemistry and Immunohistochemistry

Six hours after LPS or PBS injection, fresh placentas were dissected and fixed in 4% paraformaldehyde (PFA) (Affymetrix Inc.) for histology. Tissue was then immersed in 30% sucrose (Sigma‐Aldrich). Using Leica CM1950 cryostat (Leica Biosystems Inc.), the specimens were cut at 5 µm thickness and mounted on positively charged slides (Fischer Scientific). Placentas were cut transversally. For immunohistochemistry (IHC), slides were incubated in PBS solution containing 0.05% Triton X‐100 (Sigma‐Aldrich) and 5% normal goat serum (Invitrogen) for 30 min. Placental tissues were incubated with the M1 macrophage marker mouse anti‐iNOS antibody (1:50, Abcam), and the M2 macrophage maker rabbit anti‐CD163 antibody (1:200, Abcam) at 4°C overnight. Donkey anti‐mouse Alexa Fluor 568 fluorescent and donkey anti‐rabbit Alexa Fluor 488 fluorescent (1:500, Life Technologies) were secondary antibodies. DAPI (4′,6-diamino-2-phenylindole, Roche) was used to counterstain nuclei at a concentration of 1:5,000. All images analyzed were obtained from Zeiss AxioPlan 2 Microscope System. Placental data were obtained at the middle level. Analyses were performed with ImageJ by evaluators blinded to group identification.

### Statistical Analyses

Data were analyzed using Chi‐Square test and continuous variables using a One‐way ANOVA with Tukey post‐HSD test or Kruskal‐Wallis test with Bonferroni‐Dunn correction. Continuous data were tested for normality, and outliers were identified using Grubb’s test. When outliers were identified in data following normal distribution, they were excluded from the analysis. Statistical significance was set at *p* < 0.05, with consideration to multiple comparisons. Data analyses were performed with GraphPad Prism version 8 (GraphPad Software).

## Results

### Preparation and Characterization of Dendrimer-NAC Conjugate

As described previously, we successfully prepared, characterized, and validated the DNAC conjugate and the drug release mechanism for DNAC(Kurtoglu et al., 2009; Kannan et al., 2012; Mishra et al., 2014; Lei et al., 2017). Briefly, we used Fmoc protection/deprotection chemistry to functionalize hydroxyl - terminated generation-4 poly amidoamino (PAMAM) dendrimer with reactive amine groups, and then used suitable thiol-reactive pyridyldithio propionates to react with NAC to get DNAC conjugates. ^1^H NMR and high-pressure liquid chromatography (HPLC) were used to verified conjugate purity. Each dendrimer-conjugated contains 20 molecules of NAC to get a drug payload of ∼ 15 wt%. The DNAC conjugate is readily soluble in water, saline or PBS (pH 7.4). This indicated that the use of a disulfide linker enabled rapid release of NAC from the conjugate, but only when it was exposed to intracellular GSH-rich environment. We previously shown that DNAC localizes only to the site of inflammation and the component and conjugate are safe for maternal administration (Lei et al., 2017).

### Maternal Administration of DNAC Altered Cytokines Produced Following IUI Exposure

Firstly, we found that DNAC treatment does not affect dam weight, fetal weight and placental weight (data not shown). Then, we performed quantitative reverse transcription PCR (RT-qPCR) to measure the expression of pro-inflammatory mediators, interleukin (IL) -1β and nitrous oxide systems (Nos) 2, and anti-inflammatory mediator, Il-10, in the placenta of E17 at 6 h after PBS or PBS LPS exposure. The mRNA expression of *Il1β*, *Nos2* and *Il10* in the placenta were significantly increased in LPS + PBS group compared to PBS + PBS group (Figures 1B–D, *p* < 0.05), which was similar to our previous study (Na et al., 2021). Maternal administration of DNAC significantly decreased the pro-inflammatory cytokines mRNA of *Il1β*, *Nos2* in LPS + DNAC group compared to LPS + PBS group (Figures 1B,D, *p* < 0.05). This is seen in conjunction with an increase in the expression of anti-inflammatory mediator, *Il10,* in LPS + DNAC group compared to LPS + PBS group (Figure 1C). Treatment of the surgical control with DNAC (PBS + DNAC group) did not show any significant difference from the vehicle group (Figures 1B–D).

### Maternal Administration of DNAC Prevented CD45^+^ Leukocytes Infiltration in the Placenta Following IUI

**FIGURE 2:**
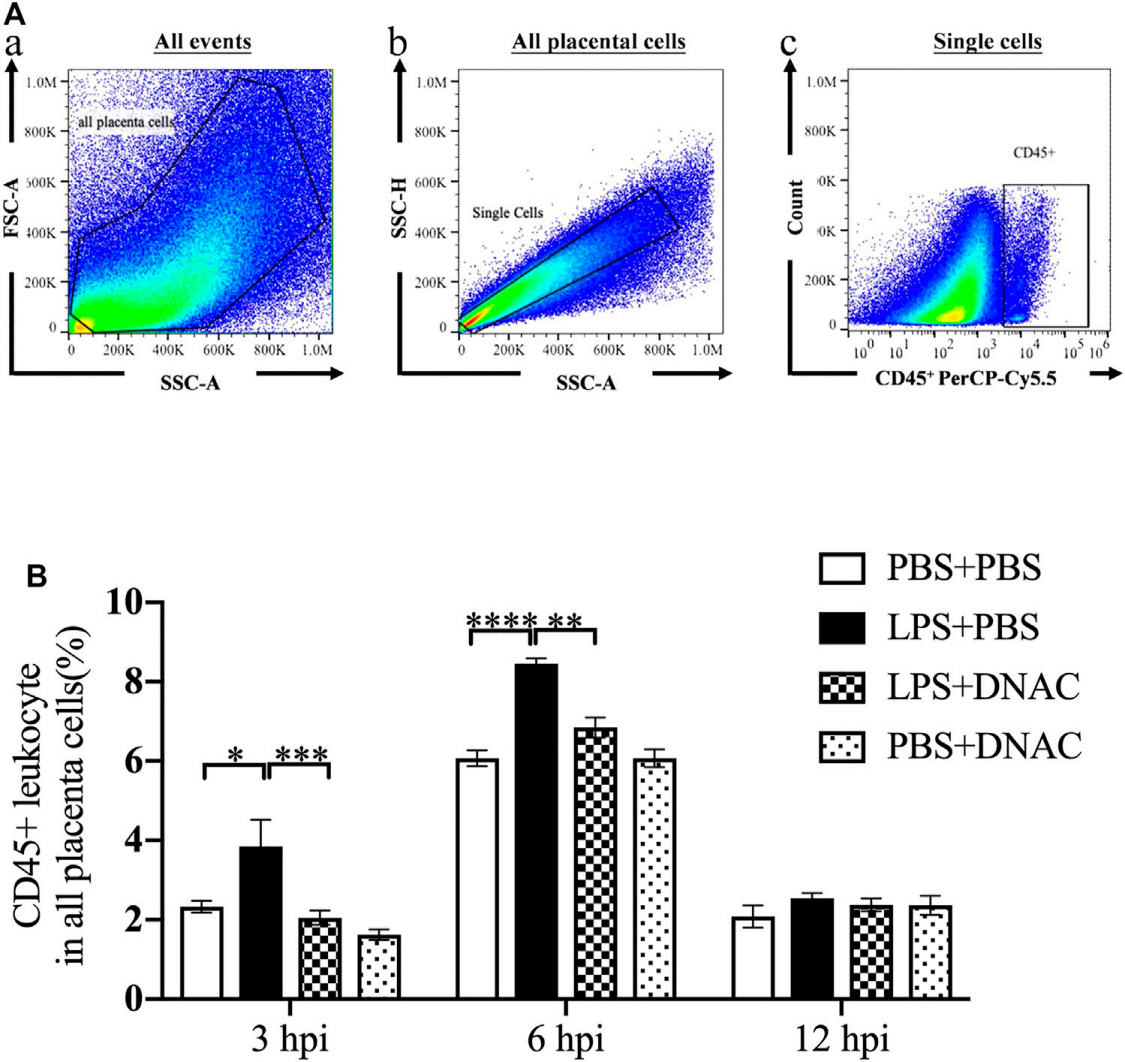
Placenta leukocytes infiltration following exposure to intrauterine inflammation and maternal Dendrimer-based N-Acetyl Cysteine (DNAC) treatment. Lipopolysaccharide (LPS) or phosphate‐buffered saline (PBS) was injected intrauterine at embryonic day 17 (E17). DNAC was injected intraperitoneally 1 h post inflammation (hpi). Placentas were collected at 3, 6 and 12 hpi. **(A)** a. All captured placental cells were distinguished based on properties of side scatter area (SSC-A) versus forward scatter area (FSC-A) generally, by using polygon/rectangular/oval gates. b. Debris and doublets were excluded by gating on side scatter height (SSC-H) versus SSC-A to further delaminate singlets. c. Placental leukocytes were gated sequentially on CD45 ^+^ versus SSC-A properties. **(B)** The ratio of CD45^+^ leukocytes infiltrated in the placenta were statically compared between groups. PBS + PBS, *n* = 16; LPS + PBS, *n* = 16; LPS + DNAC, *n* = 15; PBS + DNAC, *n* = 12. *p < 0.05 **p < 0.01 ***p < 0.001 ****p < 0.0001. Data were reported as Mean ± SEM.

Following the observation that maternal DNAC regulates cytokines expression in the placenta, we performed flow cytometry to analyze CD45^+^ leukocytes infiltration in the placenta at E17 (Figure 2A). At 3hpi and 6hpi, CD45^+^ leukocyte infiltrates in the placenta were significantly increased in LPS + PBS group compared to PBS + PBS group, while maternal treatment of DNAC significantly decreased CD45 ^+^ leukocytes infiltration in LPS + DNAC group compared to LPS + PBS group (Figure 2B, *p* < 0.05). Similarly, as our prior study demonstrated, CD45^+^ leukocyte infiltration in placenta increased with LPS-exposure at 6hpi of E17 (Lei et al., 2017; Liu et al., 2021). At 12hpi, CD45 ^+^ leukocytes infiltration in the placenta did not show any significant difference between groups (Figure 2B).

### Maternal Administration of DNAC Suppressed M1 and Increased M2 Macrophages Recruitment to the Placenta Following IUI

**FIGURE 3:**
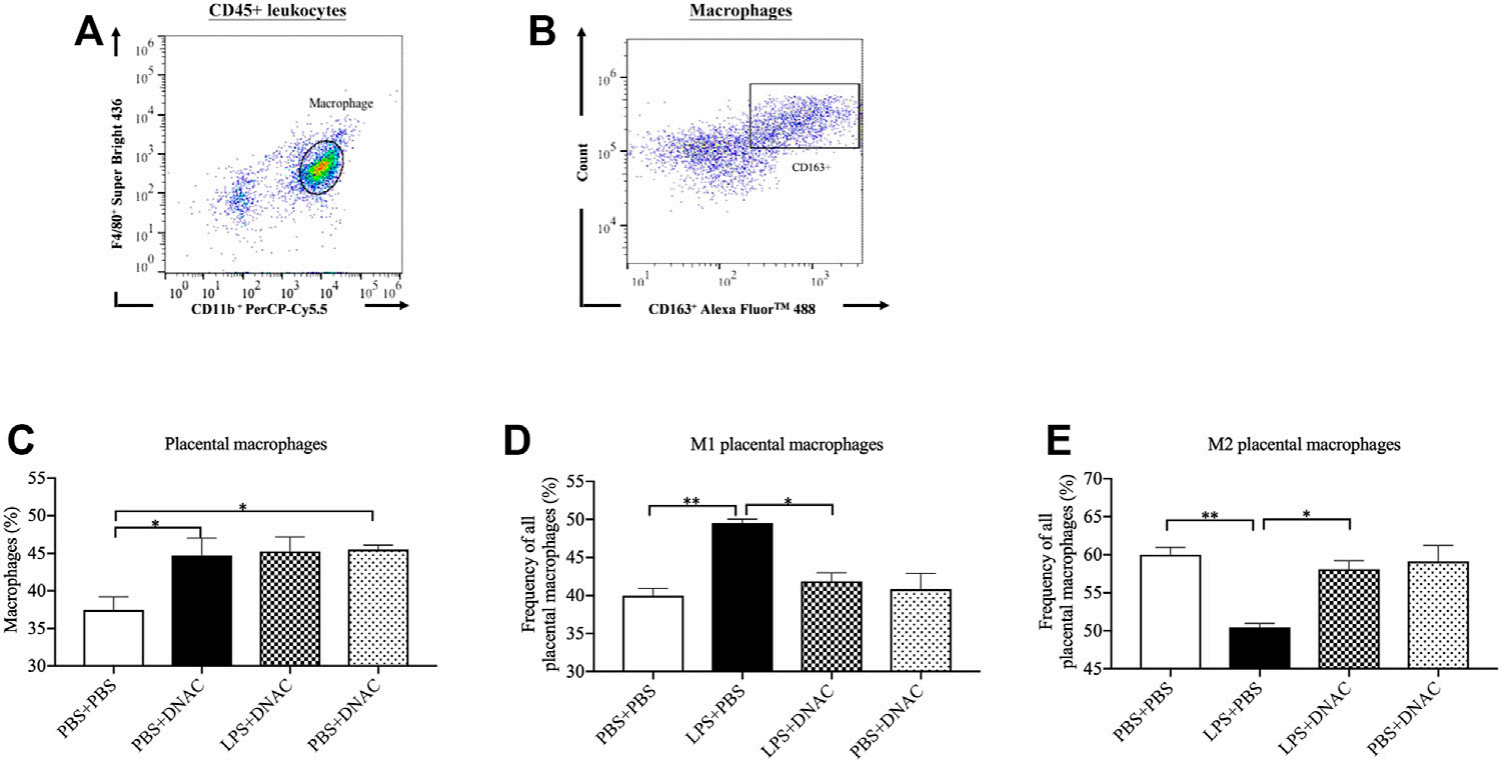
Placenta M1 and M2 phenotype macrophage polarization following exposure to intrauterine inflammation and maternal dendrimer-based N-Acetyl Cysteine (DNAC) treatment. At embryonic (E) day 17, pregnant CD-1 mice underwent a mini-laparotomy in the lower abdomen for intrauterine injection of lipopolysaccharide (LPS). One hour later, dams received intraperitoneally injection of DNAC or phosphate-buffered saline (PBS). Placentas were collected at 6 hpi after LPS or PBS injection. **(A)** The placental macrophages were further identified by F4/80+ and CD11b + properties on placental leukocytes. **(B)** M2 macrophages was identified by CD163+ and side scatter sequentially on CD45^+^ CD11b^+^ F4/80^+^ macrophages. The ratios of total macrophages **(C)**, M1 **(D)** and M2 **(E)** macrophages infiltrated in the placenta were statically compared between groups. PBS + PBS, *n* = 8; LPS + PBS, *n* = 8; LPS + DNAC, *n* = 7; PBS + DNAC, *n* = 6. *p < 0.05; **p < 0.01. Data were reported as Mean ± SEM.

Our previous research has shown that placental macrophages are the primary cells producing IL-1β, iNOS and IL-10 (Na et al., 2021). M1 macrophages express CD80, CD86, CCR7 and CD40, while M2 macrophages express CD163 and CD206 on the cell surface (Sinha et al., 2005; Xu et al., 2006; Tardito et al., 2019; Heusinkveld et al., 2011; Rocher et al., 2012). We found that maternal DNAC treatment significantly decreased the leukocytes infiltration in placenta following exposure to LPS. We further used flow cytometry to analyze macrophage frequency for the placenta 6 h after PBS or LPS exposure at E17 (Figures 3A,B). The number of placental macrophages increased significantly with LPS exposure at 6hpi (Figure 3C, *p* < 0.05), which match our prior data (Na et al., 2021). Maternal treatment with DNAC did not lead to a significant change in macrophage numbers when compared to the untreated LPS groups. However, upon further assessment of the subtypes of macrophages by flow cytometry, we found that M1 phenotype macrophages increased significantly and M2 phenotype macrophages decreased significantly in LPS + PBS group compared to PBS + PBS group (Figures 3D,E, *p* < 0.05). Furthermore, maternal DNAC significantly decreased the M1 phenotype and increased the M2 phenotype macrophages in the LPS + DNAC group when compared to LPS + PBS group (Figures 3D,E, *p* < 0.05).

### DNAC Treatment Alleviated M1 Macrophages and Increased M2 Macrophages Infiltration in Placenta Following IUI

**FIGURE 4:**
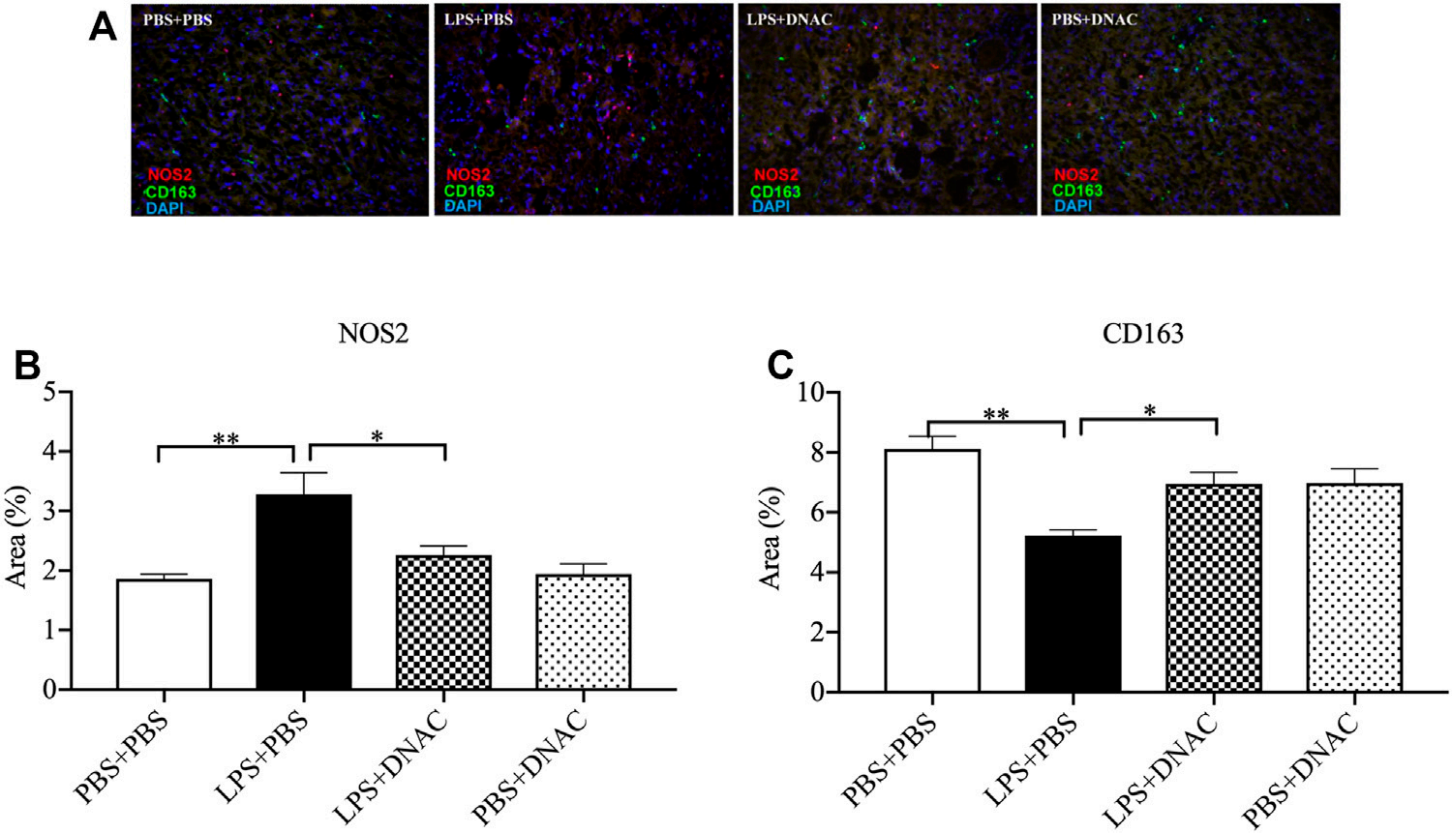
Immunohistochemical staining (IHC) of M1 and M2 macrophages in the placenta following exposure to intrauterine inflammation and maternal dendrimer-based N-Acetyl Cysteine (DNAC) treatment. At embryonic (E) day 17, pregnant CD-1 mice underwent a mini-laparotomy in the lower abdomen for intrauterine injection of lipopolysaccharide (LPS). One hour later, dams received intraperitoneally injection of DNAC or phosphate-buffered saline (PBS). Placentas were collected at 6 hpi after LPS or PBS injection. Placentas were stained with anti-NOS2 (red, a marker for M1 macrophages) and anti-CD163 (green, a marker for M2 macrophage) antibodies. DAPI (blue) staining identified the nuclei. **(A)** Representative image of IHC. NOS2+ **(B)** and CD163+ **(C)**cells were quantified in the labyrinth of the placenta and were statically compared between groups. Images are ×40 magnification, and scale bars represent 50 μm *n* = 5 for each group. *p < 0.05 **p < 0.01. Data were reported as Mean ± SEM.

We further performed IHC staining for iNOS and CD163 in placental tissue 6 h after PBS or LPS exposure at E17 (Figure 4A). IHC staining for iNOS demonstrated significant increased in iNOS expression in the LPS + PBS group compared to PBS + PBS, while maternal treatment with DNAC decreased iNOS expression when compared to LPS + PBS (Figure 4B, *p* < 0.05). IHC staining for CD163 demonstrated decreased CD163 expression in LPS + PBS group compared to the PBS + PBS group, while maternal administration with DNAC significantly increased CD163 expression in LPS + DNAC group compared to LPS + PBS group (Figure 4C, *p* < 0.05). No significant differences were seen in iNOS or CD163 staining between PBS + PBS and PBS + DNAC groups (Figures 4B,C).

## Discussion

We investigated the effect of maternal DNAC therapy towards ameliorating placental inflammation through macrophage recruitment and polarization *via* M1/M2, following IUI. We found that DNAC decreased CD45^+^ leukocytes infiltration in the placenta following IUI. DNAC altered placental immune profile, which stimulated macrophages to polarize to the M2 phenotype while decreasing the M1 phenotype, thus suppressing the inflammatory responses in the placenta. Specifically, our results demonstrated that maternal DNAC significantly reduced the pro-inflammatory cytokines *Il1β* and *Nos2* following IUI.

Hydroxyl dendrimer nanoparticles have several structural benefits, including reduced toxicity and interactions with proteins due to its hydroxyl end groups and near-neutral charge, specific targeting and uptake by “activated” macrophages, rapid cell entry, and rapid elimination of dendrimer through the kidney with minimal uptake in off-target organs (Menjoge et al., 2010; Magro et al., 2020). We explored the mechanism of action of maternal DNAC on placental inflammation treatment. D-NAC conjugates were stable at physiological conditions and without releasing NAC throughout 24 ∼ 72 h (Kurtoglu et al., 2009; Kannan et al., 2012; Nance et al., 2017). Our data provided evidence that maternal treatment with a low dose of dendrimer-conjugated NAC (DNAC-10 mg/kg of NAC) inhibits CD45^+^ leukocytes infiltration in the placenta at 3hpi and 6hpi. These data and the previously published data support the role of DNAC in enhancing anti-inflammatory response in the placenta following IUI.

Inducible nitric oxide synthase (iNOS) is a crucial enzyme in the inflammatory response of macrophages and is a source of nitric oxide (NO) that effectively induces inflammatory stimulation (MacMicking et al., 1997). IL-1β elevated by inflammation during pregnancy is one factor that causes immunological, histological, and biochemical changes in the placenta (Tsimis et al., 2017; Lee et al., 2019a; Novak et al., 2019) and damaged fetal brain (Chudnovets et al., 2020). In this study, we identified that DNAC treatment suppressed the LPS-induced production of pro-inflammatory cytokines (iNOS and IL-1β). In our previously study, we provide evidence that DNAC suppresses inflammatory response in the placenta through decreased IL-6 and tumor necrosis factor (TNF)-α following IUI. Maternal intrauterine infection associated with increased levels of pro-inflammatory cytokines (IL-6, TNF-α, IL-1β) is the most important cause of preterm birth and neonatal neurological disorders (Duggan et al., 2001; Goepfert et al., 2004; Girard et al., 2010). These results suggested that low dose of DNAC improved maternal and placental inflammatory status by dampening the pro-inflammatory pathway caused by IUI.

Successful pregnancy depends on regulating dynamic and highly regulated immunologic processes (Mor et al., 2011), especially the placenta, in which macrophages play a critical role. Macrophages are involved in all states of all pregnancy, and the M1/M2 dynamics appropriate balance (Zhang et al., 2017). M1 polarized macrophages are more effective in clearing antigens and converting T cell responses into T helper-1 (Th1) immune responses. In contrast, M2 subsets have immunosuppressive capabilities that help tissue remodeling and promote T helper- 2 (Th2) or antibody-mediated immune response (Mantovani et al., 2004; Varin and Gordon, 2009; Zhang et al., 2017). M1 macrophage phenotype markers iNOs and CXCL10 are related to pro-inflammatory cytokines (IL-1β, IL-6), M2 macrophage phenotype markers MR2 and TREM2 are related to anti-inflammatory cytokines (IL-4, IL- 10). In our study, LPS increased IL-1β mRNA expression and M1 macrophages infiltration in the placenta, these data match our previous studies (Na et al., 2021). Furthermore, we found that DNAC prevents M1 macrophage and cytokinesis iNOs and IL-1ꞵ expression. Nevertheless, in our studies, DNAC decreased M1 macrophages and increased M2 macrophage infiltration in the placenta following IUI. These data further provide evidence that DNAC maintains M1/M2 to anti-inflammation in the placenta.

In both humans and mice, immune cells are critical to maintaining tissue homeostasis, defending against infection, and contributing to labor initiation. The maternal-fetal interface supports multiple cellular immune systems comprising adaptive immune T cells and homeostatic innate immune cells such as macrophages. T cells have been shown to participate in placental pathological inflammation during pregnancy (Lee et al., 2011; Arenas-Hernandez et al., 2019; Liu et al., 2021). We proved that DNAC prevents CD3^+^/CD45^+^ cells increased by LPS-induced placental inflammations, but free NAC treatment does not. DNAC decreased CD8^+^ T-cell infiltrates in LPS-induced inflammation. Macrophages interact with T cells to regulate T cell activation and infiltration in target organs (Chellan et al., 2013). These findings concluded that DNAC initiates innate (T cells) and acquired immunity (macrophages) to play pivotal roles in protecting placenta inflammation flowing IUI.

Placental macrophage infiltration following IUI has a strong correlation with fetal microglia activation (Na et al., 2021). Based on these results, we believed that DNAC administration prevented the macrophage polarization in placental inflammatory load, which indirectly benefits fetal neuroinflammation. As demonstrated in our previous studies, DNAC improved neurobehavioral profiles and decreased microglial activation in offspring (Lei et al., 2017).

Clinically, prevention and treatment options are limited, especially in human preterm deliveries. Most therapies focus on antibiotics, considering the association between inflammation and premature labor in humans. Other new treatment strategies for PTB include progesterone (Almutairi et al., 2021; Frey et al., 2021), tocolytics (Zheng et al., 2017; Jameson and Bernstein, 2019), anti-inflammatory agents (Chin et al., 2016; Xu et al., 2016; Gorasiya et al., 2018; Lee et al., 2019b) and high throughput screening (Le et al., 2020) for PTB prophylaxes (Zierden et al., 2021). In additional to the novel drugs, new drug delivery systems such as nanomedicine-based systems, help the drugs with more targeted and sustained approach. In our previous study, we checked the component and conjugate safety of DNAC. Most clinical therapies for preterm birth focus mainly on the maternal side and have limited benefits for the fetus. In this study, we found that DNAC treatment does not affect dam weight, fetal weight and placental weight. Besides, our previous study demonstrated that dendrimer-conjugated treatment decreased IUI-induced preterm birth rate, reduced placenta and fetal brain inflammation, and improved fetal neuroinflammation in offspring (Lei et al., 2017). This study further provide evidence that DNAC has advantages in preventing placental inflammation following IUI.

In summary, we provide evidence that DNAC regulates the polarization of placenta macrophages into M1 macrophages and induces the polarization of placenta macrophages into M2 macrophages, thus suppressing the placental inflammatory responses caused by intrauterine inflammation. Our results suggest a novel mechanism for DNAC to prevent intrauterine inflammation in placenta immune activation. DNAC shows potential as an experimental treatment for preventing inflammation-related perinatal period in clinical trials.

## Data Availability

The original contributions presented in the study are included in the article/Supplementary Material, further inquiries can be directed to the corresponding author.
